# The Importance of an Interdisciplinary Approach in the Transition of At-Risk Patients From Hospital to Skilled Nursing Facilities: A Case Report

**DOI:** 10.7759/cureus.71629

**Published:** 2024-10-16

**Authors:** Michelle Y Ko, Navid Darouian

**Affiliations:** 1 Medicine, David Geffen School of Medicine, University of California Los Angeles, Los Angeles, USA; 2 Internal Medicine, University of California Los Angeles, Los Angeles, USA

**Keywords:** elder patients, interdisciplinary team, skilled nursing facility, transitional care, transition of care

## Abstract

Significant gaps exist in the transition of care for patients from acute hospitalization to skilled nursing facilities. Amongst the several well-studied interventions that exist to improve the transition of care, few investigate the efficacy of using an interdisciplinary team (IDT) approach. Here, we report the case of a 78-year-old man with a complicated social situation who had a successful medical and social transition of care from the hospital to the skilled nursing facility (SNF) with an IDT meeting that entailed the collaboration of hospital and SNF physicians and social workers.

## Introduction

Approximately 23.9% of Medicare beneficiaries make the transition from acute hospitalization to a skilled nursing facility (SNF), with trends demonstrating an increasing percentage over the years, highlighting the importance of a high-quality transition of care for this population that is potentially more vulnerable given their older age (≥65 years) and thus increased likelihood of having age-related medical comorbidities and limited social resources [1]. Ineffective transitions of care result in a lack of continuity of care, which can result in redundancy and frustration for patients and their caregivers, as well as avoidable adverse events, such as adverse drug events, follow-up errors for diagnostic tests, and nosocomial infections, which can result in rehospitalizations. In fact, approximately 23% of Medicare beneficiaries discharged to an SNF are reported to be readmitted to the hospital within 30 days, an alarming statistic given that readmissions are associated with a quadrupled mortality rate within six months [2-4]. Current data on the transition of care from hospitals to SNFs is limited, as the majority of studies focus instead on interventions that improve the transition from hospital to home. Furthermore, these studies largely focus on interventions led by or focused on single disciplines, such as the implementation of medication reports at discharge by hospital physicians, protocols for a minimum number of home follow-up visits by advanced nurse practitioners, patient counseling and follow-up by pharmacists, and even educational interventions directed at patients and their caregivers to take more active roles in transitions of care [2]. Something that has been far less well studied is the outcomes associated with the use of an interdisciplinary team (IDT) meeting approach intervention to transition patients from hospitals to SNFs. Here, we report the case of a 78-year-old man with a complicated social situation who had a successful medical and social transition of care from the hospital to the SNF with an IDT meeting composed of hospital and SNF physicians and social workers.

## Case presentation

A 78-year-old man was brought to the emergency room (ER) after being found unresponsive in his apartment by neighbors, who had not seen him for several days. Previously, the patient had been under regular care from his primary care physician but had been lost to follow-up for the past three years. He lived alone, independently managing his activities of daily living without external support.

Upon arrival at the ER, the patient reported a mechanical fall and an inability to get up afterward. His workup revealed a positive COVID-19 test, acute kidney injury, malnutrition, and concerns of adult failure to thrive, prompting hospital admission for further medical management. Examination during the admission was fairly unremarkable except for his neurological examination with memory deficits noted. This was further investigated with a cognitive assessment revealing impairment, with a Montreal Cognitive Assessment (MoCA) score of 22. Discussions with the patient’s closest living relatives disclosed that he had been estranged from his family for over 20 years, with no other relatives or friends involved in his life. Neurology was consulted given the concerns with magnetic resonance imaging (MRI) of the brain demonstrating chronic ischemic changes consistent with vascular dementia.

After receiving treatment for his acute medical conditions, the patient was deemed stable for discharge to an SNF, following recommendations from the hospital’s physical therapy and occupational therapy teams. Recognizing his high risk for poor outcomes as a vulnerable patient with newly diagnosed vascular dementia, the healthcare team decided to hold an IDT meeting prior to discharge. This meeting held over a conference call included the hospitalist, the accepting SNF physician, hospital case management, the SNF social worker, and the patient’s niece, ensuring a coordinated transition of care. It was determined that the patient would be best served by discharge to the SNF, where the facility's team would initiate the conservatorship process through Los Angeles County, considering his limited support system. The team members from the facility were able to ask clinically relevant questions to the inpatient team regarding the aspects of the evaluation already completed and the recommendations for the next steps once admitted to the facility. The family member voiced appreciation for the collaboration between both sides and expressed reassurance regarding the plan formulated during the meeting.

## Discussion

IDTs are composed of groups of two or more disciplines that collaborate, with formal and informal communication, joint problem solving, and overlap of roles for the common goal of providing good care for the patient [5-6]. IDTs are commonly utilized for the care of SNF patients, who are often older and with more comorbidities, including cognitive impairment [3]. One systematic review studied randomized controlled trials (RCTs) of IDT interventions on nursing home residents and found that of the 27 RCTs identified, the majority (66%, n = 18/27) demonstrated statistically positive effects [7]. These studies investigated a wide scope of IDT interventions, such as those directed toward psychiatric care and activities, blood pressure medications, and exercise activities, and measured several primary outcomes, including psychotropic reduction, depression and behaviors, use of restraints, number of prescriptions, incidence of falls, and more [7]. Successful interventions tended to include the participation of the patient’s primary physician and/or pharmacists, team communication, and team coordination, all of which were present in the IDT transition of care meeting held for the patient in this case [7]. Our IDT meeting also included the patient’s family members and the hospital and SNF social worker, who were particularly important to include given the patient’s complex social situation and need for conservatorship. Notably, few if any studies sought to research the potential impact of IDT-focused interventions on transitional care gaps, despite this being a potentially well-suited approach to an area of great need [7].

Poor transitions of care have been implicated in errors and adverse events, leading to hospital readmissions. Areas of gaps in transitions of care have been identified by qualitative studies. In one study, hospital and SNF clinicians identified eight of the 10 domains of the Ideal Transitions of Care Framework (ITCF) with significant gaps, including discharge planning; complete communication of information; availability, timeliness, clarity of organization and information; medication safety; educating patients to promote self-management; enlisting the help of social and community supports; coordinating care among team members; and managing symptoms after discharge [8]. More specifically, clinicians identified gaps to include issues with closed-loop communication, cross-facility coordination after patients are admitted to SNFs, and variability in quality and completeness of records transferred, including rehabilitation documentation [8]. Another multiple-case study interviewed staff, patient, and family caregiver perspectives on transitional care from hospitals to SNFs. Here, a major gap that was identified was the confinement of interactions between individual patients and individual staff members during the delivery of transitional care, which thus limited potential benefits that could have resulted from a collaboration of a larger care team. Specifically, study participants also noted that lack of team member interactions meant care teams were not always aware of all members, including family members, that were involved [9]. Several of the gaps mentioned in these studies could potentially be addressed with the use of an IDT team meeting and approach.

Our report highlights a case where an IDT meeting played a crucial role in facilitating the smooth medical and social transition of care for a high-risk patient from an acute care hospital to an SNF. The meeting also ensured continuity and clarity regarding the initiation of the conservatorship process through LA County. By organizing a conference call with representatives from the hospital, SNF, and the patient’s family, the team created an open forum to address any questions or concerns prior to the transition. This collaborative discussion involved a thorough review of diagnostic findings and critical social aspects of the case, with direct input from the patient’s family members. As a result, the team developed a detailed, step-by-step plan to ensure a seamless transition to the SNF, minimizing potential lapses in care that might have otherwise occurred without this coordinated effort.

Though clinical examples and clinical intuition might suggest the benefit of an IDT approach to transitional care for high-risk patients, further studies are needed to quantify its efficacy. IDT-based interventions remain difficult to investigate given the lack of consensus for how to define IDTs. IDTs have been shown to vary widely in terms of organizational structure, team relationships, and sensemaking, or how members make sense of what is happening with patients, all of which are factors that have been associated with differences in patient outcomes such as readmission rates [10]. Additionally, effective IDT meetings may be individualized and vary according to patient-specific needs, such as was the case of our patient with a complex social situation whose IDT meeting involved hospital and SNF social workers. Furthermore, as IDT meetings require the time and participation of multiple healthcare members, they may be best utilized as a resource reserved for at-risk patients, a definition that may be guided by scoring systems such as the LACE Index [11]. The LACE Index, which stands for length of stay (L), acuity of admission (A), comorbidities (C), and recent emergency department use (E), has been used before to predict patients at high risk of readmission within 30 days [11]. Our patient had a LACE score of 12, which put him at high risk for readmission. Ultimately, future studies aiming to investigate IDT approaches to the transition of care from the hospital to SNFs should carefully consider appropriate target populations and the potential for individualized yet well-defined IDT approaches.

## Conclusions

Significant gaps remain in transitional care from acute hospitalizations to skilled nursing facilities, such as with closed-loop communication and cross-facility coordination, resulting in patient frustrations and avoidable adverse outcomes. There are few studies investigating the efficacy of an IDT approach to transitional care, despite the successful use of IDT approaches in other aspects of nursing home activities. Clinicians should consider advocating for an IDT approach to facilitating successful and smooth transitions of care for at-risk patients, given that this approach emphasizes team and collaborative approaches to patient care that could reduce redundancy, improve clear communication, and result in more comprehensive and holistic care.
